# Cancer potencies and margin of exposure used for comparative risk assessment of heated tobacco products and electronic cigarettes aerosols with cigarette smoke

**DOI:** 10.1007/s00204-020-02924-x

**Published:** 2020-10-06

**Authors:** Gregory Rodrigo, Guy Jaccard, Donatien Tafin Djoko, Alexandra Korneliou, Marco Esposito, Maxim Belushkin

**Affiliations:** PMI R&D, Philip Morris Products S.A., Rue des Usines 56, 2000 Neuchâtel, Switzerland

**Keywords:** Margin of exposure approach, Cancer potency, Heated tobacco product, Risk assessment, Electronic cigarette

## Abstract

Health risk associated with the use of combustible cigarettes is well characterized and numerous epidemiological studies have been published for many years. Since more than a decade, innovative non-combusted tobacco products have emerged like heated tobacco products (HTP) or electronic cigarettes (EC). Long-term effects of these new products on health remain, however, unknown and there is a need to characterize associated potential health risks. The time dedicated to epidemiological data generation (at least 20 to 40 years for cancer endpoint), though, is not compatible with innovative development. Surrogates need, therefore, to be developed. In this work, non-cancer and cancer risks were estimated in a range of HTP and commercial combustible cigarettes based upon their harmful and potentially harmful constituent yields in aerosols and smoke, respectively. It appears that mean lifetime cancer risk values were decreased by more than one order of magnitude when comparing HTPs and commercial cigarettes, and significantly higher margin of exposure for non-cancer risk was observed for HTPs when compared to commercial cigarettes. The same approach was applied to two commercial ECs. Similar results were also found for this category of products. Despite uncertainties related to the factors used for the calculations and methodological limitations, this approach is valuable to estimate health risks associated to the use of innovative products. Moreover, it acts as predictive tool in absence of long-term epidemiological data. Furthermore, both cancer and non-cancer risks estimated for HTPs and ECs highlight the potential of reduced risk for non-combusted products when compared to cigarette smoking.

**Electronic supplementary material:**

The online version of this article (10.1007/s00204-020-02924-x) contains supplementary material, which is available to authorized users.

## Introduction

Electronic cigarettes (EC) and heated tobacco products (HTP) are becoming popular alternatives to cigarettes in a number of countries. For example, HTPs have partially replaced cigarettes in Japan since their introduction (Stoklosa et al. 2019), while the prevalence of ECs has risen in the United States (Berry et al. 2018; Soneji et al. 2018) and in a number of other countries (Filippidis et al. 2017). EC aerosols are generated from the heating of liquids (Farsalinos and Polosa 2014), and HTP aerosols are generated from the heating of tobacco (Smith et al. 2016) at temperatures far below the combustion temperatures observed for cigarettes. As a consequence, lower concentrations of harmful and potentially harmful constituents (HPHC) in their aerosols are measured when compared with mainstream smoke from reference (Bekki et al. 2017; Goniewicz et al. 2014; Li et al. 2019; Margham et al. 2016; Nicol et al. 2020; Schaller et al. 2016) or commercial (Jaccard et al. 2017; Mallock et al. 2018) cigarettes. An average reduction in concentrations of more than 90% among given lists of HPHCs have been observed in the aerosol of commercial HTPs or ECs against their concentrations in 3R4F reference cigarette or commercial cigarette smoke (Forster et al. 2018; Jaccard et al. 2017; Margham et al. 2016; Schaller et al. 2016).

The aerosol chemical characterization is only one part of the full product assessment of HTPs and ECs (Murphy et al. 2017; Peitsch et al. 2018) as potential reduced risk alternatives to continued cigarette smoking. The full assessment of such products includes other pre-clinical tests, such as in vitro assays, in vivo testing in animal models, and clinical studies (Murphy et al. 2017; Smith et al. 2016). Effectively, an average mean reduction against HPHCs measured in cigarette smoke does not represent a corresponding reduction of risk for a consumer switching from cigarettes to HTPs or ECs. This would imply that all HPHCs have the same potencies, which is not true. Approaches have, therefore, been developed that are aimed to estimate the risk associated with the use of these products on the basis of the individual constituents to which a consumer is exposed to.

A method for comparing the impact on carcinogenicity of tobacco products has been recently published (Slob et al. 2020). Considering the yields of specific compounds in the emissions of evaluated products, the authors utilized the resulting change in cumulated emission (CCE) to assess the impact on health effects in a semiquantitative way. The described model is based on relative potency factors (RPFs) estimated from animal data and the benchmark dose approach. After derivating the CCE from the RPFs for the considered compounds, the CCE value is translated into an estimate of the health impact. According to the authors, a CCE greater than 10 may indicate harm reduction, whereas a CCE lower than 1 would suggest an increase in harm. A CCE of 1 would be regarded as no change for the health impact. While the proposed model is valid, this method is limited to the availability of carcinogenicity study data to estimate RPF corresponding to the considered compounds.

In a recent publication, the risk associated to the exposure of a mixture composed of selected mainstream cigarette smoke constituents was determined using specific risk algorithms (Pack et al. 2018). The incremental lifetime cancer risk (ILCR) was used to determine the cancer risk and the hazard quotient (HQ) was calculated to evaluate the non-cancer risk. These two descriptors indicated that inhalation of the selected constituents have the potential to increase both cancer and non-cancer health risk.

More specifically, some authors (Chen et al. 2017; Hahn et al. 2014; Lachenmeier et al. 2018; Stephens 2017) have recently performed quantitative risk assessments to better characterize the impact of the decrease of individual HPHCs in ECs and HTPs on risk when compared to cigarettes. Stephens (2017) used cancer potency values to characterize the cancer risk, while the non-cancer hazard of ECs (Chen et al. 2017; Hahn et al. 2014) and HTPs (Lachenmeier et al. 2018) was evaluated with the margin of exposure (MOE).

The MOE approach has also been used to segregate and prioritize individual HPHCs in cigarette mainstream smoke (Cunningham et al. 2011; Haussmann 2012; Xie et al. 2012). Cunningham et al. (2011) reached the conclusion that acrolein and formaldehyde are the compounds to consider in priority for exposure reduction research. More generally, the MOE approach has been applied for the safety assessment of genotoxic and carcinogenic impurities in food and feed products (Benford 2016; Committee 2012) or to evaluate the risks associated with pesticides (Boobis et al. 2008).

The cancer potency approach has also been previously used for carcinogen prioritization in cigarette smoke (Fowles and Dybing 2003). The authors showed that the contribution of chemicals was different according to the source of considered cancer potency factors. Using those from the U.S. Environmental Protection Agency (EPA), arsenic ranked first, followed by acetaldehyde and 1,3-butadiene. Considering the thresholds published by the California Office of Environmental Health Hazard Assessment (OEHHA, the State of California EPA), 1,3-butadiene was the main contributor to the calculated cancer risk index, more than twice that of acrylonitrile, the next highest contributing carcinogen in this case. Arsenic then ranked third.

Cancer risk assessment based on the cancer potency approach has also been used for substances or products other than tobacco. For example, lung cancer risk in the general population following exposure to zinc cadmium sulfide was estimated from Army testing according to this approach (National Research Council Subcommittee on Zinc Cadmium 1997). Cancer potency was considered to characterize cancer risk after exposure to pesticides like chlorpyrifos and dichlorvos (Kim et al. 2013) or to food contaminants (Vogt et al. 2012).

Our analysis combined MOE and cancer potency values with the goal of quantifying cancer and non-cancer risks associated with exposure to HPHCs from a range of commercial HTPs and ECs when compared with those from reference and commercial cigarettes on the basis of available compound specific toxicological threshold references from official regulatory agencies.

## Materials and methods

### Sample selection

Samples of commercial cigarettes and HTPs were purchased between 2017 and 2019 at the point of sale. Two hundred and seventy-three cigarette brands and eight HTP brands from various manufacturers were included in this study. Electrically heated and aerosol-heated HTPs were considered (eHTP and aHTP, respectively) (CORESTA 2020), as both types are commercially available. In the absence of full HPHC datasets for EC aerosols, the results published for two commercial closed EC systems (Margham et al. 2016; Nicol et al. 2020) were used.

### HPHC yield determination

The analyses of HPHCs were performed at Labstat International (Kitchener, Ont., Canada) under contract. For cigarettes, the applied analytical smoking machine regime followed the specifications of the ISO intense smoking regime (ISO 2018a) modifications. For eHTPs, the same regime was used for the generation of their aerosols, because it is the regime that has been most widely used for such products (Belushkin et al. 2018) and is the regime currently recommended for their assessment (CORESTA 2020). The puff number per product unit was determined. Puff numbers ranged from 6.1 to 13.4 for cigarettes and from 8 to 12 for stick-type HTPs. For aHTP and EC aerosol generation, the regime described in the CRM 81 (CORESTA 2015) and in ISO 20,768 (ISO 2018b) was used, with a horizontal orientation of the products on the vaping machine. The determination of HPHCs in cigarette smoke and HTPs was performed according to Labstat accredited internal methods, as previously described (Jaccard et al. 2017). Forty-two HPHCs were measured in HTP aerosols and 33 HPHCs were measured in cigarette mainstream smoke. The concentration (C; µg/100 mL) in aerosol and in smoke was reported for each analyzed HPHC, according to the Table 3 below. The difference in terms of number of measured HPHC is only due to a larger list of HPHC applied to the analysis of HPHC.

In the absence of proprietary datasets related to a full list of EC emissions, the HPHC yields published by Margham et al. (2016) and by Nicol et al. (2020) were compiled. For comparison purposes, the HPHCs considered for the mean lifetime cancer risk and for the MOE evaluations of ECs were the same as those considered for HTPs.

The results below the limit of quantification (LOQ) for individual HPHCs in EC or HTP aerosols were replaced by the LOQ values. If the values were below LOQ for all product categories for a given HPHC, this compound was not evaluated further.

### Cancer potency and mean lifetime cancer risk determination

Cancer potencies of emissions from either HTP aerosol, EC aerosol, or cigarette smoke were determined as described by Stephens (2017) with slight modifications. Briefly, inhalation unit risks (IUR) published by the California Office of Environmental Health Hazard Assessment (OEHHA) or the U.S. Environmental Protection Agency (EPA) were selected, whichever was the highest. IURs refer to the increased cancer risk associated with lifetime exposure to 1 µg of substance per m^3^ air inhaled (EPA 2005). IURs were considered for each constituent analyzed in HTP, EC, or cigarette emissions and classified by the International Agency for Research on Cancer (IARC) as known (IARC 1), probably (IARC 2A), or possibly (IARC 2B) carcinogenic to human.

Chromium is classified differently by IARC depending on its speciation. Indeed, hexavalent chromium or Cr(VI) is classified IARC 1, whereas trivalent and metallic chromium (Cr(III) and Cr(0), respectively) are classified IARC 3 (not classifiable as to its carcinogenicity to humans). It has previously been reported that Cr(VI) is not the one mobilized in cigarette smoke (Campbell et al. 2014; Fresquez et al. 2017). Chromium was, therefore, not included in the cancer potency determination.

Isoprene was evaluated by IARC in 1994, reevaluated in 1999 (IARC 1999), and is classified 2B. Quinoline was evaluated by IARC in 2019 (IARC 2019) and is classified 2B. However, no reliable IUR, neither for isoprene nor for quinolone, was available, which fulfilled our inclusion criteria. Isoprene and quinolone were, therefore, not included in the cancer potency determination.

According to these criteria and the availability of HPHC yield data for HTP aerosols and smoke from commercial cigarettes, 13 compounds were selected for cigarettes and 21 were selected for HTPs and ECs (Table 1).

**Table 1:** List of compounds analyzed in cigarettes, HTPs, and ECs and their unit risk considered for cancer potency determination

Compound	IARC type	IUR (µg/m^3^)^−1^	IUR Origin	Cigarettes	HTPs	ECs
Acetaldehyde	2B	2.7 × 10^–6^	OEHHA	✓	✓	✓
Formaldehyde	1	1.3 × 10^–5^	EPA	✓	✓	✓
1,3-Butadiene	1	1.7 × 10^–4^	OEHHA	✓	✓	✓
Acrylonitrile	2B	2.9 × 10^–4^	OEHHA	✓	✓	✓
Arsenic	1	4.3 × 10^–3^	EPA	✓	✓	✓
Cadmium	1	4.2 × 10^–3^	OEHHA	✓	✓	✓
Nickel	2B	2.6 × 10^–4^	OEHHA	✓	✓	✓
Vinyl chloride	1	7.8 × 10^–5^	OEHHA		✓	✓
Benzene	1	2.9 × 10^–5^	OEHHA	✓	✓	✓
Benzo[a]pyrene	1	1.1 × 10^–3^	OEHHA	✓	✓	✓
Acrylamide	2A	1.3 × 10^–3^	OEHHA		✓	✓
Ethylene oxide	1	3.0 × 10^–3^	EPA		✓	✓
Propylene oxide	2B	3.7 × 10^–6^	EPA		✓	✓
2-Aminonaphthalene	1	5.1 × 10^–4^	OEHHA	✓	✓	✓
4-Aminobiphenyl	1	6.0 × 10^–3^	OEHHA	✓	✓	✓
o-Toluidine	1	5.1 × 10^–5^	OEHHA		✓	✓
Lead	2B	1.2 × 10^–5^	OEHHA	✓	✓	✓
N-Nitrosonornicotine (NNN)	1	4.0 × 10^–4^	OEHHA	✓	✓	✓
Benz[a]anthracene	2B	1.1 × 10^–4^	OEHHA		✓	✓
Acetamide	2B	2.0 × 10^–5^	OEHHA		✓	✓
Dibenz[a,h]anthracene	2A	1.2 × 10^–3^	OEHHA		✓	✓

*IARC* International Agency for Research on Cancer, *OEHHA* California Office of Environmental Health Hazard Assessment, *EPA* U.S. Environmental Protection Agency, *IUR* inhalation unit risk, *HTP* heated tobacco product, *EC* electronic cigarette

The cancer potency related to product emissions was then calculated and translated to mean lifetime cancer risk, considering a daily aerosol intake (DAI) based on a daily consumption (DC) of 20 cigarettes, 20 sticks for stick-type HTPs, or 20 L of inhaled aerosol for liquid-based HTPs or ECs and a daily breathed volume (DBV) of 20 m^3^. The mean lifetime cancer risk refers to the estimated risk of developing cancer due to exposure to a product aerosol incurred over the lifetime of an individual.As the product emissions were generated using the ISO intense smoking regime (ISO 2018a) for cigarettes and stick-type HTPs, the puff volume was fixed to 55 mL. The calculations were performed according to the below formula, *i* referring to the ith product and *j* to the jth compound:

$$ {\text{Cancer potency}}_{i}  = \sum\limits_{{j = 1}}^{n} {IUR_{j} C_{{i,j}} } , $$

$${\text{Lifetime cancer risk}}_{{\text{i}}}  = \frac{{DAI_{i} }}{{DBV}} \times {\text{cancer potency}}_{i} , $$

For cigarettes and stick-type HTPs, DAI was determined as follows:

$${DAI}_{i}=\mathrm{puff volume}\times {\mathrm{puff number}}_{\mathrm{i}}\times {DC}_{i},$$

For liquid-based HTPs and ECs, DAI was determined as follows:

$${DAI}_{i}=0.001\times {DC}_{i}.$$

### MOE determination

MOEs were determined as described by Baumung et al. (2016) and Lachenmeier et al. (2018) with slight modifications. Briefly, for a given compound, its MOE is defined as the ratio of its toxicity effect level (the toxicological threshold of the compound) to its estimated human exposure:

$$\mathrm{MOE}=\frac{\mathrm{Toxicological} \mathrm{threshold}}{\mathrm{Human estimated exposure dose}} ,$$

the toxicological threshold being either NOAEL, LOAEL, BMDL, or a derived reference dose or concentration (ADI, RfD, RfC, DNEL …). Where applies, MOE is then corrected by uncertainty factors, as defined by regulatory agencies (ECHA 2012; EPA 2002). MOE are usually used for risk characterization to determine if health risk may occur under specific exposure scenarios. The lower the MOE, the more likely a chemical is to pose an unreasonable risk. In a comparative assessment, the product with the highest MOE is considered at lower risk.

In this context, inhalation exposure limits (IEL) made publically available by the European Chemical Agency (EChA), OEHHA, or EPA were selected as toxicological threshold, whichever was the lowest. Derived no effect levels (DNEL) were retrieved from EChA. DNELs are toxicological exposure limits based on exposure scenarios established by manufacturers for chemical substances in accordance with Registration, Evaluation, Authorisation and Restriction of Chemicals regulation (EU 2006). DNELs should contribute to protect populations against adverse health effects from chemical exposure. Reference exposure levels (REL) were retrieved from OEHHA. RELs are concentration levels at or below which inhalation exposure to the human population is likely to be without an appreciable risk of deleterious effects during a specified exposure duration (OEHHA 1999). Reference concentrations (RfC) were retrieved from EPA. RfCs are concentrations provided by the Integrated Risk Information System assessment program (EPA 2002). They are derived by estimating a continuous inhalation exposure with no anticipated adverse health effects to the human population (including sensitive subgroups) during a lifetime. DNELs, RELs or RfCs are already corrected by uncertainty factors and applying new uncertainty factors to IELs is, therefore, judged unnecessary. IELs were considered for each constituent analyzed in HTP or cigarette emissions and upon availability. According to these criteria, 29 compounds were selected for cigarettes, and 32 were selected for HTPs and ECs (Table 2).

**Table 2:** List of compounds analyzed in cigarettes and HTPs and their exposure limit considered for MOE determination

Compound	IEL (mg/m^3^)	IEL Origin	Cigarettes	HTPs	ECs
Acetaldehyde	9.0 × 10^–3^	EPA	✓	✓	✓
Acrolein	2.0 × 10^–5^	EPA	✓	✓	✓
Formaldehyde	9.0 × 10^–3^	OEHHA	✓	✓	✓
1,3-Butadiene	2.0 × 10^–3^	EPA	✓	✓	✓
Acrylonitrile	2.0 × 10^–3^	EPA	✓	✓	✓
Arsenic	1.5 × 10^–5^	OEHHA	✓	✓	✓
Cadmium	2.0 × 10^–5^	OEHHA	✓	✓	✓
Chromium	2.0 × 10^–4^	OEHHA	✓	✓	✓
Mercury	3.0 × 10^–5^	OEHHA	✓	✓	✓
Nickel	1.4 × 10^–5^	OEHHA	✓	✓	✓
Vinyl chloride	2.0 × 10^–3^	ECHA		✓	✓
Ammonia	2.0 × 10^–1^	OEHHA	✓	✓	✓
Hydrogen cyanide	9.0 × 10^–3^	OEHHA	✓	✓	✓
Benzene	3.0 × 10^–3^	OEHHA	✓	✓	✓
Toluene	3.0 × 10^–1^	OEHHA	✓	✓	✓
Methyl ethyl ketone	5.0 × 10^0^	EPA	✓	✓	✓
m-Cresol	7.5 × 10^–1^	ECHA		✓	✓
o-Cresol	7.5 × 10^–1^	ECHA	✓	✓	✓
p-Cresol	7.5 × 10^–1^	ECHA		✓	✓
m + p-Cresol	7.5 × 10^–1^	ECHA	✓		
Phenol	2.0 × 10^–1^	OEHHA	✓	✓	✓
Benzo[a]pyrene	2.0 × 10^–6^	EPA	✓	✓	✓
Isoprene	8.4 × 10^0^	ECHA	✓	✓	✓
Propionaldehyde	8.0 × 10^–3^	EPA	✓	✓	✓
Acrylamide	6.0 × 10^–3^	EPA		✓	✓
Ethylene oxide	3.0 × 10^–2^	OEHHA		✓	✓
Selenium	1.5 × 10^–2^	ECHA	✓	✓	✓
Styrene	9.0 × 10^–1^	OEHHA	✓	✓	✓
Acetone	2.0 × 10^+2^	ECHA	✓	✓	✓
Propylene oxide	3.0 × 10^–2^	EPA		✓	✓
Pyridine	6.0 × 10^–1^	ECHA	✓	✓	✓
Catechol	3.0 × 10^–1^	ECHA	✓	✓	✓
Hydroquinone	1.1 × 10^0^	ECHA	✓	✓	✓
Resorcinol	1.4 × 10^0^	ECHA	✓	✓	✓
Nicotine	5.6 × 10^–3^	ECHA	✓	✓	✓

OEHHA: California Office of Environmental Health Hazard Assessment; EPA: U.S. Environmental Protection Agency; ECHA:European Chemicals Agency; IEL: inhalation exposure limits; HTP: heated tobacco product; EC: electronic cigarette

The MOEs were defined as the ratio of the IEL level to the estimated human exposure (EHE) level. IEL levels were calculated on the basis of a DBV of 20 m^3^. EHE levels were determined for each listed compound and for each evaluated product based on their emission yield and considering a daily consumption of 20 cigarettes, 20 sticks for stick-type HTPs, or 20 L of inhaled aerosol for hybrid HTPs and ECs. Two combined margins of exposure (MOE_T_) related to product emissions were then calculated, one including nicotine MOE and one excluding nicotine MOE. Lower MOE_T_ points out higher risk when exposed to their related products.

The calculations were performed according to the formula below, *i* referring to the *i*th product and *j* to the *j*th compound:

$${MOE}_{i,j}=\frac{{IEL}_{j}\times DBV}{{DAI}_{i}\times {C}_{i,j}},$$

$${MOE}_{{T}_{i} }=\frac{1}{\sum_{j=1}^{n}\frac{1}{{MOE}_{i,j}}},$$

### Statistical analysis

Nonparametric tests were performed to get an insight on the statistical difference between the median values of the three parameters of interest; mean lifetime cancer risk, MOE_T_ excluding nicotine MOE, and MOE_T_ with nicotine MOE included, between conventional cigarettes and heated tobacco products. The two-sample Wilcoxon rank sum test (Mann–Whitney test) was considered as nonparametric test with a *P* value set at 0.05. It was computed with the software R studio, version 3.6.2 (2019–12-12).

## Results

### HPHC yields

Minimum and maximal yields of 33 compounds analyzed in the smoke from 273 cigarette brands and 42 compounds in the aerosol of 8 HTPs brands sold worldwide and 2 ECs are reported in Table 3. The individual results for the eight HTPs are provided in supplementary material. The variability observed among HPHC yields is due to the various blends and cigarettes or HTP and ECs designs. To a certain extent, however, this reflects what people may be exposed to globally during product consumption. Non-quantifiable concentrations were set at the LOQ, and non-detectable concentrations were set to the limit of detection. Few concentrations were not determined (ND) in cigarette mainstream smoke or in HTP aerosol.

**Table 3:** Minimum and maximum yields of considered compounds in smoke from 273 brands of cigarettes, in aerosol from 8 brands of HTPs sold worldwide, and in aerosols from 2 ECs (µg/100 mL)

Compound	Cigarettes		HTPs		ECs	
	Min	Max	Min	Max	Min	Max
1,3-Butadiene	7.21 × 10^0^	2.44 × 10^1^	6.59 × 10^–3^	5.26 × 10^–2^	2.59 × 10^–3^	5.09 × 10^–3^
2-Aminonaphthalene	1.62 × 10^–3^	8.54 × 10^–3^	2.06 × 10^–6^	4.20 × 10^–6^	7.27 × 10^–7^	1.41 × 10^–6^
4-Aminobiphenyl	2.92 × 10^–4^	1.40 × 10^–3^	2.27 × 10^–7^	1.90 × 10^–6^	3.47 × 10^–7^	1.09 × 10^–6^
Acetaldehyde	1.72 × 10^2^	3.99 × 10^2^	6.72 × 10^–2^	3.28 × 10^1^	3.93 × 10^–2^	1.91 × 10^–1^
Acetamide	ND	ND	6.23 × 10^–2^	5.05 × 10^–1^	2.18 × 10^–3^	4.36 × 10^–3^
Acetone	6.96 × 10^1^	1.51 × 10^2^	1.12 × 10^0^	5.38 × 10^0^	1.08 × 10^–1^	1.32 × 10^–1^
Acrolein	1.89 × 10^1^	3.93 × 10^1^	3.30 × 10^–2^	1.46 × 10^0^	8.36 × 10^–3^	1.27 × 10^–1^
Acrylamide	ND	ND	2.18 × 10^–2^	2.76 × 10^–1^	5.55 × 10^–3^	1.09 × 10^–2^
Acrylonitrile	1.75 × 10^0^	6.41 × 10^0^	7.27 × 10^–3^	2.43 × 10^–2^	2.91 × 10^–3^	5.82 × 10^–3^
Ammonia	2.76 × 10^0^	1.84 × 10^1^	2.59 × 10^–1^	2.09 × 10^0^	1.13 × 10^–1^	3.99 × 10^–2^
Arsenic	1.02 × 10^–3^	3.21 × 10^–3^	5.45 × 10^–5^	1.82 × 10^–4^	3.08 × 10^–4^	3.46 × 10^–4^
Benz[a]anthracene	ND	ND	1.27 × 10^–5^	4.68 × 10^–4^	3.32 × 10^–6^	2.87 × 10^–5^
Benzene	6.29 × 10^0^	2.15 × 10^1^	3.86 × 10^–3^	8.24 × 10^–2^	1.53 × 10^–3^	3.27 × 10^–3^
Benzo[a]pyrene	1.83 × 10^–3^	5.69 × 10^–3^	2.00 × 10^–5^	1.42 × 10^–4^	4.83 × 10^–6^	9.82 × 10^–6^
Cadmium	2.28 × 10^–3^	5.01 × 10^–2^	1.36 × 10^–5^	3.64 × 10^–5^	1.85 × 10^–5^	1.49 × 10^–4^
Catechol	1.31 × 10^1^	3.29 × 10^1^	9.09 × 10^–3^	2.17 × 10^0^	2.34 × 10^–3^	4.73 × 10^–3^
Chromium	5.37 × 10^–3^	1.18 × 10^–2^	5.02 × 10^–4^	1.95 × 10^–3^	7.25 × 10^–4^	2.11 × 10^–3^
Dibenz[a.h] anthracene	ND	ND	1.88 × 10^–5^	2.82 × 10^–5^	5.63 × 10^–6^	1.82 × 10^–5^
Ethylene oxide	ND	ND	7.27 × 10^–3^	3.47 × 10^–2^	3.26 × 10^–3^	6.55 × 10^–3^
Formaldehyde	1.07 × 10^1^	4.02 × 10^1^	8.15 × 10^–2^	1.76 × 10^0^	7.64 × 10^–2^	2.22 × 10^–1^
Hydrogen cyanide	3.52 × 10^1^	9.98 × 10^1^	9.45 × 10^–2^	3.98 × 10^–1^	2.38 × 10^–2^	4.76 × 10^–2^
Hydroquinone	1.10 × 10^1^	3.17 × 10^1^	1.41 × 10^–2^	1.09 × 10^0^	5.66 × 10^–3^	1.13 × 10^–2^
Isoprene	5.04 × 10^1^	1.96 × 10^2^	9.32 × 10^–3^	2.27 × 10^–1^	3.69 × 10^–3^	7.27 × 10^–3^
Lead	3.92 × 10^–3^	2.35 × 10^–2^	4.25 × 10^–5^	3.69 × 10^–4^	6.29 × 10^–5^	4.18 × 10^–4^
m + p-Cresol	1.30 × 10^0^	6.41 × 10^0^	ND	ND	ND	ND
m-Cresol	ND	ND	1.36 × 10^–3^	4.32 × 10^–3^	5.14 × 10^–4^	1.09 × 10^–3^
o-Cresol	5.28 × 10^–1^	2.70 × 10^0^	1.82 × 10^–3^	8.59 × 10^–3^	7.02 × 10^–4^	5.38 × 10^–2^
p-Cresol	ND	ND	2.27 × 10^–3^	7.73 × 10^–3^	9.35 × 10^–4^	1.82 × 10^–3^
Mercury	4.29 × 10^–4^	2.68 × 10^–3^	3.82 × 10^–5^	3.98 × 10^–4^	1.90 × 10^–6^	8.22 × 10^–5^
Methyl ethyl ketone	1.64 × 10^1^	4.08 × 10^1^	1.05 × 10^–1^	1.15 × 10^0^	4.73 × 10^–3^	1.26 × 10^–1^
Nickel	5.85 × 10^–3^	1.29 × 10^–2^	6.89 × 10^–4^	3.77 × 10^–3^	1.12 × 10^–3^	1.28 × 10^–3^
Nicotine	2.16 × 10^2^	5.03 × 10^2^	2.36 × 10^1^	1.72 × 10^2^	5.75 × 10^1^	6.55 × 10^1^
N-Nitrosonornicotine (NNN)	3.03 × 10^–3^	1.40 × 10^–1^	1.82 × 10^–5^	3.92 × 10^–3^	9.09 × 10^–6^	9.79 × 10^–5^
o-Toluidine	ND	ND	2.37 × 10^–5^	2.12 × 10^–4^	1.03 × 10^–5^	1.42 × 10^–5^
Phenol	1.51 × 10^0^	1.07 × 10^1^	5.91 × 10^–3^	2.00 × 10^–1^	2.34 × 10^–3^	2.04 × 10^–2^
Propionaldehyde	1.32 × 10^1^	2.79 × 10^1^	7.27 × 10^–2^	2.06 × 10^0^	4.36 × 10^–3^	4.85 × 10^–2^
Propylene oxide	ND	ND	3.55 × 10^–3^	2.06 × 10^–2^	1.42 × 10^–3^	2.84 × 10^–3^
Pyridine	3.43 × 10^0^	1.49 × 10^1^	9.09 × 10^–3^	1.05 × 10^0^	4.73 × 10^–3^	1.06 × 10^–2^
Resorcinol	2.16 × 10^–1^	9.90 × 10^–1^	2.42 × 10^–3^	5.45 × 10^–3^	1.50 × 10^–3^	2.91 × 10^–3^
Selenium	2.00 × 10^–3^	8.07 × 10^–3^	7.03 × 10^–5^	2.38 × 10^–4^	1.96 × 10^–5^	7.18 × 10^–5^
Styrene	1.70 × 10^0^	5.92 × 10^0^	3.64 × 10^–3^	1.60 × 10^–1^	2.18 × 10^–3^	7.01 × 10^–3^
Toluene	1.08 × 10^1^	3.42 × 10^1^	1.39 × 10^–2^	2.75 × 10^–1^	1.13 × 10^–2^	4.53 × 10^–2^
Vinyl chloride	ND	ND	9.95 × 10^–5^	2.38 × 10^–4^	5.97 × 10^–5^	1.20 × 10^–4^

*Min *minimum yield, *Max* maximum yield, *ND* not determined, *HTP* heated tobacco product, *EC* electronic cigarette

### Cancer potency and mean lifetime cancer risk

**Fig. 1:**
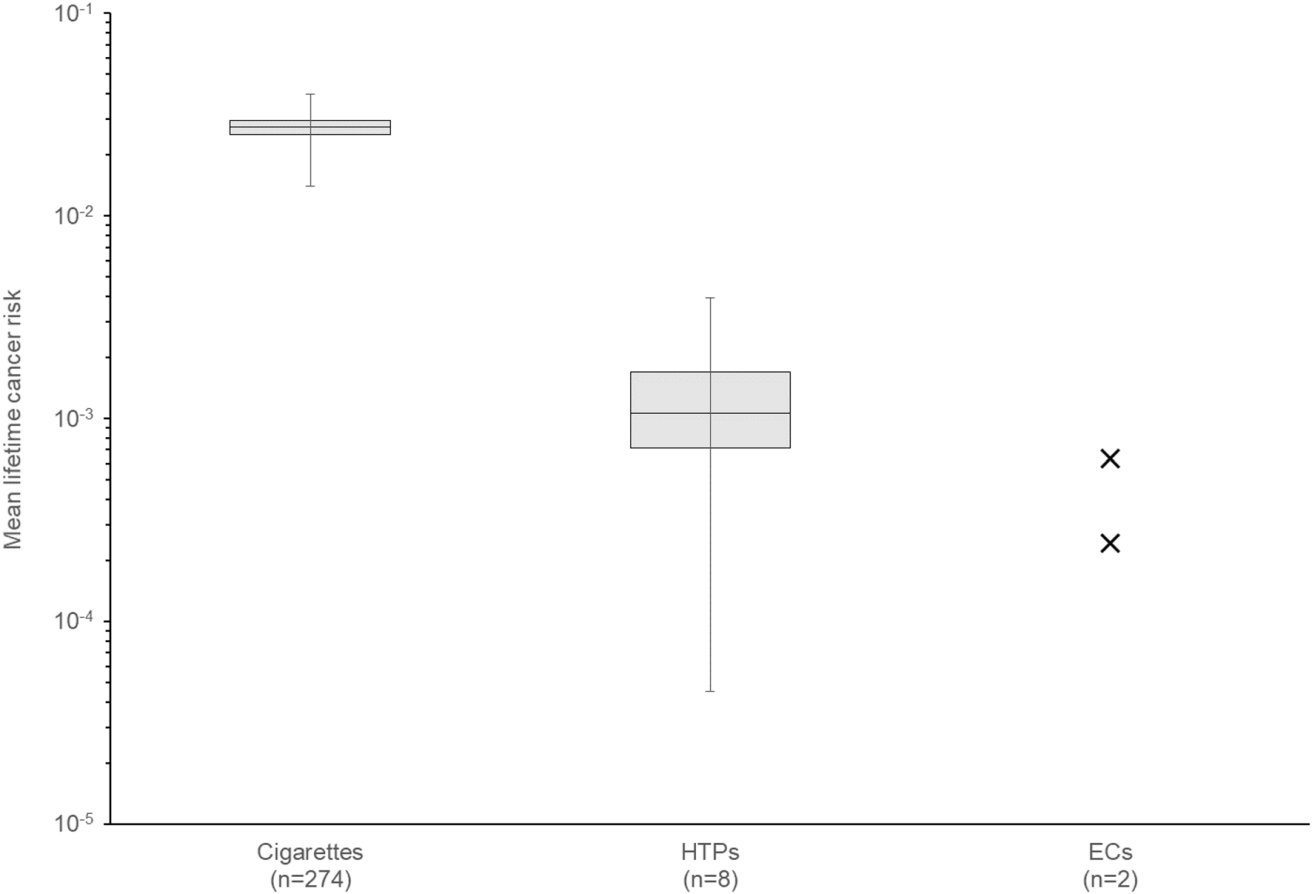
Mean lifetime cancer risk index for cigarettes and HTPs based on ISO intense smoking regime emissions and for closed-system ECs based on CRM 81/ISO 20,768 aerosol emissions

Cancer potency was determined for each selected compound and translated to mean lifetime cancer risk for each evaluated product. Mean lifetime cancer risk values were determined for cigarettes and HTPs. These values ranged from 1.40·10^–2^ to 3.97·10^–2^ for cigarettes with 2.73·10^–2^ as median. For HTP, the range extended from 4.53·10^–5^ to 3.95·10^–3^ with 1.06·10^–3^ as median. The results expressed as minimum, maximum, median, first quartile, and third quartile were plotted on a logarithmic axis (Fig. 1).

For the ECs, considering the yields published (Margham et al. 2016; Nicol et al., 2020) for the 21 selected compounds, the calculated mean lifetime cancer risk was respectively 2.42·10^–4^ and 3.95·10^–4^. As these values are representative of two products only, no statistical analysis was performed, and they were plotted as single points on Fig. 1.

Based on median analysis, the relative cancer risk for a lifetime exposure is 0.039 for exposure to HTPs compared to cigarettes. The relative cancer risk for a lifetime exposure to the considered closed-system ECs is 0.009 and 0.014 compared to lifetime exposure to cigarette smoke. This predicts a lowered cancer risk when exposed to HTP or EC aerosols compared to the exposure to cigarette smoke on the basis of the considered chemical compounds.

### MOE

**Fig. 2:**
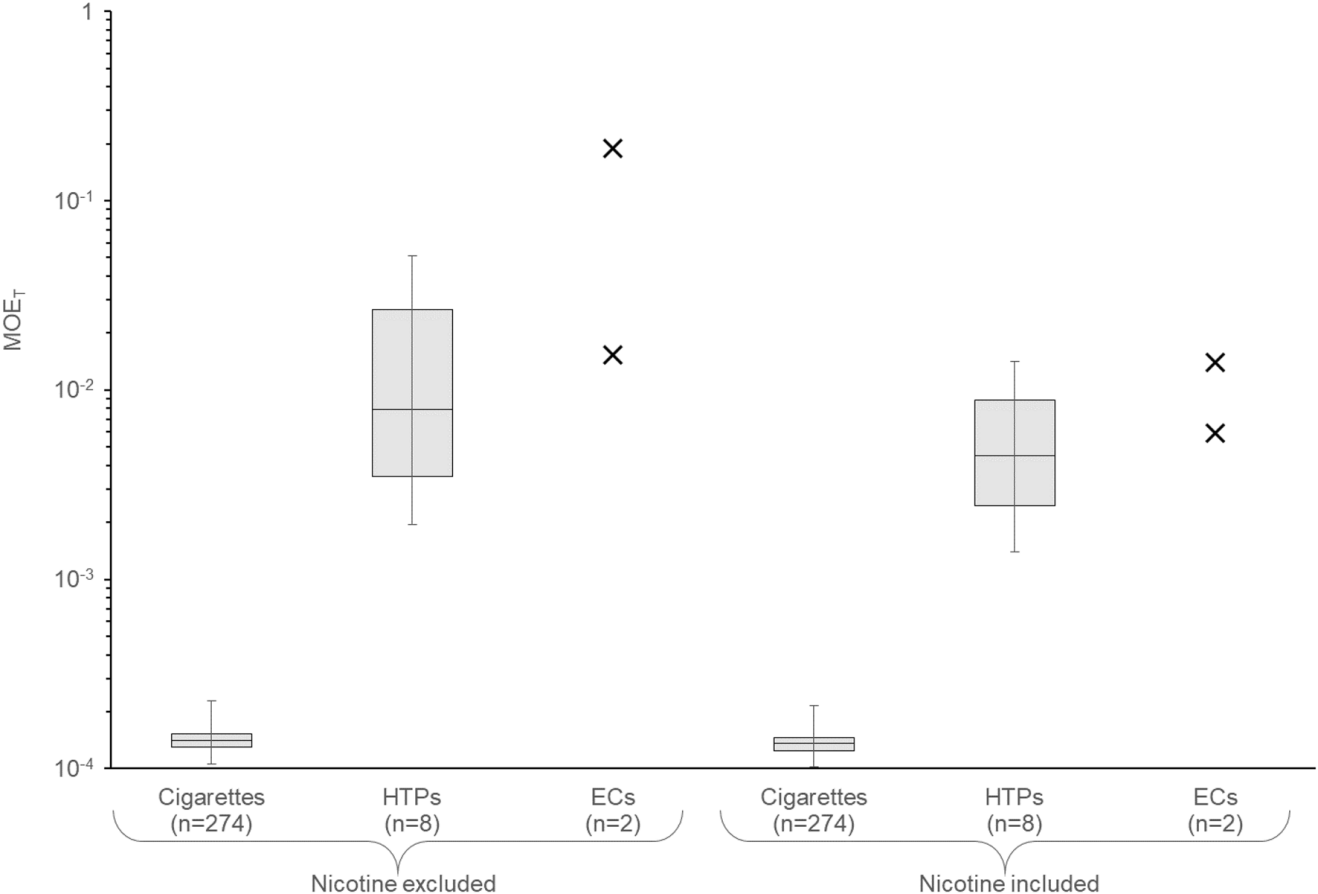
MOE_T_ for daily cigarette, HTPs, or ECs exposure excluding and including nicotine

MOE values were calculated for each of the selected compounds. MOE_T_ were then determined including or excluding the nicotine MOE for each product. MOE_T_ values (with and without considering MOE for nicotine) were determined for cigarettes, HTPs, and ECs. When MOE for nicotine was excluded, MOE_T_ values ranged from 1.06·10^–4^ to 2.28·10^–4^ for cigarettes with 1.42·10^–4^ as median. For HTPs, the range extended from 1.96·10^–3^ to 5.10·10^–2^ with 7.86·10^–3^ as median. With the inclusion of nicotine, MOE_T_ values ranged from 1.03·10^–4^ to 2.16·10^–4^ for cigarettes with 1.36·10^–4^ as median. For HTPs, the range extended from 1.40·10^–3^ to 1.42·10^–2^ with 4.49·10^–3^ as median. MOE_T_ results expressed as minimum, maximum, median, first quartile and third quartile were plotted on a logarithmic axis including or excluding MOE for nicotine (Fig. 2).

On the basis of the published EC yields for HPHCs, MOE_T_ values were 1.53·10^–2^ and 1.73·10^–1^ excluding nicotine MOE, or 5.92·10^–3^ and 8.10·10^–3^, including nicotine MOE. As these values are representative of two products only, no statistical analysis was performed, and they were plotted as single points on Fig. 2.

Median values showed an increase of the HTPs’ combined MOE (meaning an estimated reduced non-cancer risk). The relative MOE_T_ for HTPs is 55.45 when compared to cigarettes excluding nicotine and 33.05 including nicotine. The relative MOE_T_ for the considered closed-system ECs is 107.79 and 1222.12 when compared to cigarettes excluding nicotine, and 43.55 and 59.56 including nicotine. Therefore, the non-cancer risk associated with the exposure to HTP or EC aerosols is potentially reduced compared to the exposure to cigarette smoke on the basis of the evaluated chemical compounds.

### Statistical analysis

Tests on the median values were performed to examine the statistical difference between the conventional cigarettes and the heated tobacco products. Electronic cigarettes were not considered for statistical analysis due to the limited data points available. The nonparametric tests showed that at a 95% confidence level, there is evidence of significant statistical difference between the median MOE_T_ including nicotine MOE of conventional cigarettes and the median MOE_T_ including nicotine MOE of heated tobacco products. The same conclusion was obtained for the median differences between the two product categories for the mean lifetime cancer risk, and MOE_T_ excluding nicotine MOE.

## Discussion

Cigarette smoking is recognized as a major health hazard, causing diseases such as heart failure, blood vessel thrombosis, emphysema, chronic obstructive pulmonary disease, or cancer in various organs and even death (CDC 2014; WHO 2012). Smoking cessation is the most efficient way to reduce the risk of developing smoking-related diseases and early death (Anthonisen et al. 2005; CDC 1990). For legal-age smokers not willing to quit smoking, HTPs and ECs have the potential to present reduced risk when compared with continued smoking, even if a residual risk cannot be excluded (COT 2017; Nutt et al. 2014). Contrary to cigarette smoke, epidemiological data and, more generally, long-term real-life use reduced risk potential of the impact of HTPs or ECs on health are missing (Biondi-Zoccai et al. 2019; Newland et al. 2019). However, the time needed to generate epidemiological data, or even clinical data to support the assessment of relative product health risks, is not compatible with the current pace of development of novel non-combusted products. Such research can even take decades when considering endpoints such as cancer. Therefore, surrogates need to be used to overcome the lack of such data. These estimates might be regarded as insufficient for a robust conclusion of a reduced risk, because they only rely on emission data. However, these estimates do provide critical information to further develop innovative non-combusted alternatives with the potential of reduced harm when compared to continued cigarette smoking.

The health risk associated with the consumption of such non-combusted products can then be appraised by determining cancer potencies and mean lifetime cancer risk, or individual MOE and combined MOE_T_. HPHC yields in aerosols can be combined with exposure input values to estimate daily exposures. This methodology has been used previously to perform quantitative product risk assessment on cigarette smoke and HTP or EC emissions (Baumung et al. 2016; Lachenmeier et al. 2018; Stephens 2017; Xie et al. 2012).

This approach presents a few limitations. First, the impact of HPHC emission yield modulation on toxicity for specific organs cannot be determined. Effectively, MOE are not organ-specific, they have been determined as toxicological thresholds for whole organism health effect. Therefore, this methodology only allows to describe a global health risk, which serves the intended purpose of the present manuscript. Second, substances for which no IUR and/or no IEL has been previously determined cannot be included in such a risk prediction. Furthermore, potential synergistic effects between different compounds cannot be properly evaluated, as the methods rely on summing up the specific toxicity of each individual compounds. Although such limitations should be considered when assessing the results, they affect only partially the overall evaluation, and have quite a low impact when the approach is used for comparative purposes.

It is also important to acknowledge that this approach is affected by uncertainties. For example, the estimation of either the MOE or the cancer potency may be impacted by the choice of the toxicity data used to derive toxicological threshold values. Quality and reliability of the selected studies may affect the confidence of the predicted risk. To avoid this bias, our calculations are performed on the basis of exposure limits published by regulatory agencies. Even if these limits were translated from animal data, they are widely accepted among regulators and represent a reference value to determine potential health risk. The determination of HPHC emission yields constitutes an additional source of uncertainty. Effectively, the precision of the analytical methods is critical, and any deviation may distort the determination of the level for each compound considered in the aerosol. Nevertheless, the impact of the uncertainties should be balanced, as this methodology is used for comparative evaluation. By applying same uncertainties to all evaluated products, the impact of uncertainties on the predictions is reduced. In addition, this is not intended to supersede epidemiological studies and it should be regarded more as a predictive tool rather than an assessment tool, requiring therefore a lower level of precision.

Other methodologies have been published, based on ILCR and HQ (Pack et al. 2018). In this study, the cancer risk was estimated by the ILCR for each constituent, defined as follows:

$${ILCR}_{j}={IUR}_{j}\times \frac{{CY}_{j}\times \frac{RR}{100}\times \frac{(100-MS)}{100}\times NCy\times (LE-SA)}{DBV\times LE\times 365},$$

with ILCRj, the incremental lifetime cancer risk for the selected constituent; IUR_j_, the inhalation unit risk for the selected constituent (per μg/m^3^); CY_j_, the mainstream aerosol constituent yield (µg/product); RR, the respiratory retention rate (%); MS, the mouth-spill rate (%); NCy, average number of product consumed per year (product/year); LE, life expectancy; SA, age at smoking initiation; and DBV, daily breathed volume (m^3^/day). The mouth-spill rate refers to the proportion of the undiluted aerosol that can be spilled out (consciously or not) or even blown from the mouth prior to inhalation (St. Charles et al. 2013). The respiratory retention rate refers to the fraction of an aerosol constituent inhaled and retained in the respiratory tract, including both mouth and lung retention (St. Charles et al. 2013).

Compared to our approach, this methodology should be preferred only if all the parameters have been properly calculated for individual risk prediction or they fluctuate in specified ranges for probabilistic prediction. Otherwise, to estimate the risk for a general population, a conservative approach should be considered. The maximal risk will then be determined, which implies to arbitrary fix RR, MS, and SA. It assumes that RR will be fixed to 100%, meaning that the compound is completely retained in the body. In addition, MS will be fixed to 0%, meaning that the chemical is not spilled out or blown from the mouth prior to inhalation. Morever, SA may be fixed to 0, meaning that exposure is considered for lifetime. In such case, some simplification occurs with

$${ILCR}_{j}={IUR}_{j}\times \frac{{CY}_{j}\times NCy}{DBV\times 365},$$

however,

$$\frac{{CY}_{j}\times NCy}{DBV\times 365}=\frac{{C}_{j}\times {DAI}_{i}\times 365}{DBV\times 365}=\frac{{C}_{j}\times {DAI}_{i}}{DBV},$$

therefore,

$${ILCR}_{j}={IUR}_{j}\times \frac{{C}_{j}\times {DAI}_{i}}{DBV}.$$

Considering these assumptions, our approach is comparable to this methodology.

Pack et al. (2018) estimated the non-cancer risk for each constituent with the HQ, defined as:

$${HQ}_{j}=\frac{\frac{{CY}_{j}\times \frac{RR}{100}\times \frac{(100-MS)}{100}\times NCd}{DBV}}{RfC},$$

with HQ_j_, the hazard quotient for the selected constituent; CY_j_, the mainstream aerosol constituent yield (µg/product); RR, the respiratory retention rate (%); MS, the mouth-spill rate (%); NCd, average number of product consumed per day (product/day); DBV, daily breathed volume (m^3^/day); and RfC, the reference concentration for the inhalation route of the exposure (μg/m^3^).

As previously described for the cancer risk, the model described by Pack et al. (2018) for the non-cancer risk can also be used to estimate the risk for a general population. In such case, in a conservative approach and with the same assumptions mentioned above for RR and MS, some simplification occurs with

$${HQ}_{j}=\frac{\frac{{CY}_{j}\times NCd}{DBV}}{RfC}.$$

However,

$$\frac{{CY}_{j}\times NCd}{DBV}=\frac{{C}_{j}\times {DAI}_{i}}{DBV},$$

therefore,

$${HQ}_{j}=\frac{{C}_{j}\times {DAI}_{i}}{RfC\times DBV}=\frac{1}{{MOE}_{j}}.$$

Considering these assumptions, our approach is comparable to this methodology, as HQ is nothing else than the reciprocal of the proposed MOE.

Our results show a statistically significant change (*P* < 0.05) in mean lifetime cancer risk for HTPs compared to cigarette smoking. It was greatly decreased by about one order of magnitude (ratio of 0.039), with median values dropping down from 2.73·10^–2^ (cigarettes) to 1.06·10^–3^ (HTPs). In other terms, it means that 1 out of 36 smokers and 1 out of 943 HTP consumers, respectively, would develop a cancer if the cancer would only originate from the exposure to the prioritized chemicals listed in Table 1. Stephens (2017) determined a mean lifetime cancer risk of 2.4·10^–2^ and 5.7·10^–4^ for cigarette smoke and HTP aerosol with 0.024 as ratio. Even if these results are slightly different from ours, the trend is similar, showing a significant reduction in cancer risk. The differences observed are probably due to the use of different datasets for product emissions (we used eight different HTPs covering a large spectrum in terms of designs and manufacturers, while Stephens used only one product; differences in analytical methods and laboratories), the compounds used for the calculation of the estimated cancer risk, and the use of different IURs.

A more pronounced cancer risk reduction was observed when comparing the mean lifetime cancer risk for the considered ECs with that for cigarette smoke. This reduction was about two orders of magnitude (ratio of 0.009 and 0.014) with 2.42·10^–4^ and 3.95·10^–4^ for ECs compared to 2.73·10^–2^ for cigarettes. In terms of consumers, this would mean that 1 out of 36 cigarette smokers vs. 1 out of 4132 or 1 out of 2531 EC consumers may develop a cancer if the cancer root cause would be only associated with exposure to the considered HPHCs.

It should be noted that much higher yields were observed for selected aldehydes (acrolein, formaldehyde, and acetaldehyde) in a number of EC product aerosols (Belushkin et al. 2019), especially for open-system ECs. The determination, based on acetaldehyde and formaldehyde emissions only, resulted for open-system ECs in a higher estimated mean lifetime cancer risk (up to one order of magnitude) compared to the ones calculated for the considered closed-system ECs, for which a more complete emission yield dataset is available (data not shown). Nevertheless, the cancer risk was still reduced by more than one order of magnitude when comparing the mean lifetime cancer risk for the considered open-system ECs with that for cigarette smoke (ratio of 0.028, data not shown).

By analyzing the cancer potencies, it appears that different chemical compounds are the main contributors to the mean lifetime cancer risk between cigarettes and HTPs. Among the measured compounds, the comparison of their cancer potencies median showed that 1,3-butadiene, acrylonitrile, acetaldehyde, and benzene would be the main contributors for cigarettes (50.92%, 21.57%, 13.52%, and 7.29%, respectively) whereas acrylamide, ethylene oxide, acetaldehyde, and formaldehyde would principally contribute to the calculated cancer risk for HTPs (56.38%, 17.33%, 16.49%, and 4.30%, respectively), according to the followed approach. Our observations for combustible cigarettes are comparable to the results obtained by Fowles and Dybing (2003), who mentioned 1,3-butadiene, acrylonitrile, acetaldehyde, and benzene among the five main contributors. This difference in major contributors may be explained by the changes in product design between cigarettes and HTPs, in particular the much lower temperature applied to tobacco for HTPs when compared to cigarettes (Breheny et al. 2017; Smith et al. 2016; Eaton et al. 2018), which result in significant changes in product emission characteristics (Simonavicius et al. 2019). It may, however, be confusing to have acrylamide and ethylene oxide appearing as main contributors to cancer risk for HTPs, as these substances’ yields are often below LOQ in their aerosols. It could also be questionable why they do not emerge as main contributors for cigarette smoke. In the dataset analyzed for cigarette emissions, acrylamide and ethylene oxide were not determined. Mainstream smoke data are, however, available in the literature for these two compounds in the smoke of the 3R4F and 1R6F reference cigarettes (Forster et al. 2018; Jaccard et al. 2019). Applying the same methodology as described, the cancer potencies were determined for these two substances on the basis of the published yields in smoke. For these two reference cigarettes, median values of 9.58 and 101.60 were obtained for acrylamide and ethylene oxide, respectively. For HTPs on the basis of the data analyzed, cancer potency median values were 1.24 and 0.38 for acrylamide and ethylene oxide, respectively. It appears, therefore, important to balance the above results on the contribution of acrylamide and ethylene oxide to the calculated cancer risk for HTPs. Even if acrylamide and ethylene oxide seem to be main contributors to the HTP estimated mean lifetime cancer risk, individual values of cancer potency allow to evaluate the real contribution of HTP emissions compared to cigarettes.

Based on our results, a statistically significant increase (*P* < 0.05) of the combined MOE is observed when comparing HTPs to cigarette smoking. Median MOE_T_ values were increased from 1.42·10^–4^ (cigarettes) to 7.86·10^–3^ (HTPs) excluding nicotine MOE and from 1.36·10^–4^ (cigarettes) to 4.49·10^–3^ (HTPs) including nicotine MOE. The MOE_T_ for HTPs was increased by 55.45 compared to MOE_T_ for cigarettes without considering MOE of nicotine and by 33.05 when MOE of nicotine was included. Lachenmeier et al. (2018) reported a MOE_T_ increase of 23-fold excluding nicotine and tenfold including nicotine. The slight difference observed between our study and the one published by Lachenmeier et al. is mainly due to the emission constituents considered and the toxicological thresholds used to determine the MOE. In this study, exposure limits published by regulatory agencies (DNELs from EChA, REL from OEHHA, or RfC from EPA) were applied as toxicological thresholds. However, Lachenmeier et al. based their calculation on the MOE published by Baumung et al. (2016) using animal or clinical data as point of departure (no observed adverse effect level, lower limit benchmark concentration, lower limit benchmark dose, etc.). Nevertheless, the trend is conserved, which confirms that the non-cancer risk associated with the exposure to HTP aerosol is decreased compared to exposure to cigarette smoke on the basis of the considered chemical compounds (Table 2).

For the ECs, the increase of MOE_T_ was even higher. Their values were 1.53·10^–2^ or 1.73·10^–2^ with nicotine MOE excluded and 5.92·10^–3^ or 8.10·10^−3^with nicotine MOE included. Compared to those of cigarettes, the MOE_T_ were 43.55 or 59.56 times higher and even 107.79 or 1222.12 times higher when nicotine MOE was not considered. However, as previously mentioned, much higher yields were observed for selected aldehydes (acrolein, formaldehyde, and acetaldehyde) in a number of EC product aerosols (Belushkin et al 2019), especially for open-system ECs. Applying the MOE methodology (excluding nicotine) resulted in a lower estimated MOE_T_ (up to one order of magnitude) for these products compared to the ones calculated for the considered closed-system ECs, when only formaldehyde, acrolein, and acetaldehyde yields were considered in their aerosols (data not shown). Nevertheless, the MOE was still increased by about one order of magnitude when comparing the MOE_T_ for the considered open-system ECs with that for cigarette smoke (ratio of 7.964, data not shown).

In this approach to quantify the non-cancer risk associated to product emissions, nicotine appears to play a non-negligible role, especially for HTPs or ECs. Effectively, a 42.8% decrease of MOE_T_ is observed for HTPs and 61.3–95.3% is observed for ECs when nicotine MOE is taken into consideration, whereas only a 4.2% decrease is observed for cigarettes. For cigarette smoke, such a small decrease should not be considered as a much larger health risk for consumers, especially when bearing in mind the contribution of the other smoke constituents to the usual smoke-related diseases (CDC 2010). For HTPs and ECs, however, it may be justified to further investigate the impact of such decrease on consumer health risk, as the HTPs and ECs are aimed at substituting cigarettes for smokers not willing to quit. As such, they are designed to deliver nicotine to consumers with a pharmokinetic profile as close as possible to that of cigarettes to ensure that the smokers switch to the products successfully (Brossard et al. 2017; Proctor 2018; Smith et al. 2016). Baumung et al. (2016) pointed out the urgency of integrating the risk related to nicotine when assessing the health risk related to the use of nicotine-containing products (including HTPs, ECs, or smokeless tobacco). The reasoning adopted by Baumung et al. was based on the comparison of MOEs for various aerosol constituents, including nicotine. The authors concluded that nicotine risk assessment should be revised, as all MOEs they considered for nicotine were below 10 and, therefore, in the range of very high risk. Such an approach is highly dependent on the nicotine toxicological threshold chosen for MOE determination. The authors selected toxicological thresholds that are not representative of usual tobacco product consumption, which constitutes a limitation for this approach. They were either referring to nicotine acute exposure or to a different exposure route than inhalation. This may result in misestimating the risk associated with nicotine exposure from tobacco products and falsely conclude that nicotine may affect health similarly as various toxicants related to cigarette smoke exposure, with the exception of addictiveness. Nevertheless, due to the large reduction in toxicant exposure with HTPs (Simonavicius et al. 2019) or ECs (Goniewicz et al. 2017; O'Connell et al. 2016; Prokopowicz et al. 2019), it may appear legitimate to reconsider the impact of nicotine on health risk associated with the use of such products. Non-ionized nicotine is readily absorbed, independently of the administration route, and distributed throughout the body. Its acute toxicity is well characterized and directly linked to its pharmacological activity (Surgeon General 2014). Unfortunately, long-term exposure studies focusing on nicotine-specific health effects are not available and little is, therefore, known on potential health effects related to continued nicotine exposure. A few animal studies have mentioned hepatoxicity as potential adverse effect related to nicotine exposure (Azzalini et al. 2010; Salahshoor et al. 2016; Yuen et al. 1995). It is interesting to note that deleterious effects on liver were only observed after exposure to a high nicotine dose (2.5 mg/kg bw, Salahshoor et al. 2016; Yuen et al. 1995) or in pathological animals (Azzalini et al. 2010). At a lower dose (1.25 mg/kg bw, Yuen et al. 1995) or in healthy animals (Azzalini et al. 2010), liver toxicity was not observed. In addition, epidemiological studies related to the use of nicotine replacement therapies did not exacerbate clinically apparent liver injury or serum enzyme elevations (Dautzenberg et al. 2007; Marsh et al. 2005). Moreover, subchronic inhalation studies performed with nicotine-containing products showed that liver-related changes were transient and fully reverted to baseline levels at the end of a post-inhalation recovery period (Oviedo et al. 2016; Phillips et al. 2017, 2018; Wong et al. 2016). Due to their ability to revert, observed effects should probably be regarded as an adaptive response to xenobiotic exposure (Hall et al. 2012). While a few studies reported cardiovascular effects related to nicotine exposure, like increase of systolic and diastolic blood pressures as well as heart rate, thrombosis, inflammatory effects or atherosclerosis (Heeschen et al. 2001; Benowitz et al. 2002; Lee and Cooke 2011; Benowitz and Burbank 2016; Moheimani et al. 2017; Franzen et al. 2018), other concluded the absence of nicotine effect on cardiovascular system (Joseph et al. 1996; Zevin et al. 1998; Farsalinos et al. 2016; D’Ruiz et al. 2017). Whereas some studies described potential role played by nicotine in the development of lung cancer, as tumor promoter, proliferative agent, or interfering with cancer chemotherapy (Minna 2003; Grozio et al. 2007; Catassi et al. 2008; Guo et al. 2011; Kyte and Gewirtz 2018), other studies indicated that nicotine has the potential to prevent them (Kunze et al. 1999) or at least does not contribute to their occurrence (Murray et al. 2009). In vitro (Bavarva et al. 2013; Momi et al., 2013) and in vivo transgenic rodent models have been established to prove the role of nicotine in cancer progression pathways, which has however not been demonstrated in human cancer (Haussmann and Fariss 2016). Nicotine has also been suggested to impair human fertility. Effectively, reprotoxicity studies in animals showed that nicotine can significantly reduce the count and motility of sperm in rats in a dose-dependent manner (Jana et al. 2010; Oyeyipo et al. 2010). Although exposure to nicotine induced morphological changes in spermatozoa and decrease in libido in male rats, nicotine withdrawal reversed this effect (Oyeyipo et al. 2011). In addition, ex vivo exposure of sperm from healthy non-smokers showed a disparity with the animal results. Effectively, sperm needed to be exposed to concentrations far beyond physiological exposure to mimic effects observed in animal studies (Oyeyipo et al. 2014; Jorsaraei et al. 2008). Altogether, these data suggest that repeated exposure to nicotine does not represent a major issue in human with no underlying pathology. In the view of these controversial data, it would be key for consumers of HTPs or ECs to further characterize the risk associated with chronic nicotine exposure, and, if any, determine an IEL level related to potential chronic health effects other than addictiveness. This will further help in estimating the risk associated with chronic nicotine exposure more accurately.

When assessing cancer risk, guidance documents advise that acceptable lifetime cancer risk levels generally do not exceed 10^–4^, with risk levels lower than 10^–6^ considered as negligible (ECHA 2012; EPA 2005; WHO 2008). According to our approach for evaluating non-cancer risk, a MOE less than 1 is generally considered of concern for potential non-cancer effects, as the EHE level is then greater than the IEL level. On the contrary, a MOE greater than 1 reflects a non-threatening hazard for health to the global population, which also covers sensitive subpopulations. For all categories of evaluated products, the lifetime cancer risk level exceeded 10^–4^, and the MOE_T_ was less than 1. Cancer and non-cancer risks, as estimated in this study, support therefore that none of these products are risk-free. Nonetheless, HTPs and ECs showed a large decrease in estimated cancer and non-cancer risks in comparison with the predicted cancer and non-cancer risks induced with cigarettes, highlighted by a large reduction of the mean lifetime cancer risk index (around 25 times for HTPs and 69–112 times for ECs) and a major increase of the MOE_T_ with and without including the nicotine MOE (around 33 and 55 times for HTPs, 43–59 and 107–1222 times for ECs, respectively). HTPs and ECs may therefore appear as a justifiable alternative for legal-age smokers unable or unwilling to quit smoking.

## Conclusions

HTPs and ECs are commercially available alternatives to cigarettes. While the cancer and the non-cancer risk associated with cigarette smoke is well characterized, this is not the case for the aerosol from HTPs or ECs. No long-term epidemiological data currently exist to determine potential health risks associated with the use of such products. Due to the observed growing numbers of smokers who adopt these non-combusted alternatives, surrogates need to be developed to estimate the health risk associated with the use of these reduced exposure products. Our approach was based on the use of emission yields to determine mean lifetime cancer risk index and combined MOE_T_. Indicators obtained from HTPs and ECs were compared to those from cigarettes, and a quantitative product risk assessment was performed. This methodology has some limitations, mainly the selection of specific HPHCs and the availability of IURs or IELs for the considered HPHCs, as well as the criteria developed for their selection. The chemical characterization of HTP and EC aerosols is still an ongoing activity. Compounds with potential toxicological concern may have been ignored, because the current analytical technology did not allow their detection. Even if they should not be considered as risk-free products, however, HTPs and ECs lead to an appreciable risk reduction in comparison to cigarettes, both for cancer and non-cancer diseases. According to the current knowledge, and more specifically to the data presented here, HTPs and ECs might be considered as an acceptable reduced risk substitute for cigarettes for legal-age smokers who would otherwise continue smoking cigarettes.

## Electronic supplementary material

Below is the link to the electronic supplementary material.

Supplementary file1 (PDF 237 kb)
