# Supplementary material for: Cancer potencies and margin of exposure used for comparative risk assessment of heated tobacco products and electronic cigarettes aerosols with cigarette smoke
Source: Arch Toxicol. 2020 Oct 6;95(1):283–98. doi: 10.1007/s00204-020-02924-x (PMC7811518; doi:10.1007/s00204-020-02924-x)
Supplement: Supplementary file 1 — Supplementary file1 (PDF 237 kb) [file 204_2020_2924_MOESM1_ESM.pdf]

Table 4: List of evaluated HTP brands

Product name	Product technology	Product manufacturer	Consumable	Puffs per consumable
**Lil**	Heated Tobacco	KT & G	Fiit Change (KR) - Uncrushed Capsule	12
**Glo**	Heated Tobacco	British American Tobacco	Kent Neosticks Brights Tobacco	8
**Lil Plus**	Heated Tobacco	KT & G	Fiit Sparky	8
**Ploom S**	Heated Tobacco	Japan Tobacco	Mevius Menthol	8
**Lil Hybrid**	Hybrid	KT & G	Miix Golden Taste	8
**Ploom Tech**	Hybrid	Japan Tobacco International	MEVIUS Regular	50
**Ploom Tech +**	Hybrid	Japan Tobacco International	Mevius Mild Blend	50
**IQOS**	Heated Tobacco	Philip Morris International	Heets	12

Table 5: HPHC yields in evaluated HTP brands (µg/100 mL)

Compound	A	B	C	D	E	F	G	H
**1,3-Butadiene**	5.26×10^{-2}	6.59×10^{-3}	2.13×10^{-2}	5.18×10^{-2}	1.09×10^{-2}	7.27×10^{-3}	1.09×10^{-2}	6.59×10^{-3}
**1-Amino naphthalene**	6.14×10^{-6}	1.82×10^{-6}	4.09×10^{-6}	ND	ND	7.88×10^{-6}	ND	6.14×10^{-6}
**2-Amino naphthalene**	ND	ND	ND	4.20×10^{-6}	ND	ND	2.06×10^{-6}	ND
**4-Aminobiphenyl**	8.33×10^{-7}	2.27×10^{-7}	5.56×10^{-7}	1.45×10^{-6}	ND	1.90×10^{-6}	2.42×10^{-7}	1.36×10^{-6}
**Acetaldehyde**	2.84×10^{1}	6.80×10^{0}	2.02×10^{1}	3.28×10^{1}	6.72×10^{-2}	1.76×10^{1}	2.89×10^{-1}	9.30×10^{0}
**Acetamide**	2.34×10^{-1}	1.26×10^{-1}	2.48×10^{-1}	5.05×10^{-1}	ND	3.59×10^{-1}	6.23×10^{-2}	1.94×10^{-1}
**Acetone**	3.81×10^{0}	1.50×10^{0}	2.81×10^{0}	5.38×10^{0}	ND	1.24×10^{0}	ND	1.12×10^{0}
**Acrolein**	1.19×10^{0}	5.41×10^{-1}	8.89×10^{-1}	1.46×10^{0}	3.30×10^{-2}	5.40×10^{-1}	5.27×10^{-2}	1.62×10^{-1}
**Acrylamide**	9.55×10^{-2}	5.16×10^{-2}	1.56×10^{-1}	2.76×10^{-1}	ND	2.15×10^{-1}	2.18×10^{-2}	9.53×10^{-2}
**Acrylonitrile**	2.43×10^{-2}	7.27×10^{-3}	1.62×10^{-2}	2.39×10^{-2}	ND	7.27×10^{-3}	1.09×10^{-2}	7.27×10^{-3}
**Ammonia**	1.49×10^{0}	9.82×10^{-1}	1.24×10^{0}	1.59×10^{0}	ND	5.78×10^{-1}	2.59×10^{-1}	2.09×10^{0}
**Arsenic**	8.18×10^{-5}	1.45×10^{-4}	5.45×10^{-5}	1.82×10^{-4}	6.36×10^{-5}	8.18×10^{-5}	6.36×10^{-5}	8.18×10^{-5}
**Benzene**	7.06×10^{-2}	3.86×10^{-3}	4.14×10^{-2}	8.24×10^{-2}	ND	1.27×10^{-2}	1.70×10^{-2}	3.86×10^{-3}
**Benz[a] anthracene**	2.35×10^{-4}	5.52×10^{-5}	1.88×10^{-4}	3.15×10^{-4}	ND	4.68×10^{-4}	1.27×10^{-5}	1.41×10^{-4}
**Benzo[a]pyrene**	ND	ND	ND	1.42×10^{-04}	2.00×10^{-05}	ND	2.00×10^{-05}	ND
**Catechol**	8.08×10^{-1}	1.95×10^{-2}	6.79×10^{-1}	2.17×10^{0}	ND	7.53×10^{-1}	9.09×10^{-3}	4.42×10^{-2}

**Cadmium**	2.05×10^{-5}	3.48×10^{-5}	1.36×10^{-5}	4.24×10^{-5}	3.64×10^{-5}	2.00×10^{-5}	3.64×10^{-5}	2.05×10^{-5}
**Chromium**	1.33×10^{-3}	7.52×10^{-4}	5.02×10^{-4}	5.02×10^{-4}	8.86×10^{-4}	1.92×10^{-3}	9.70×10^{-4}	1.95×10^{-3}
**Dibenz[a,h] anthracene**	2.82×10^{-5}	2.82×10^{-5}	1.88×10^{-5}	1.88×10^{-5}	ND	2.73×10^{-5}	2.18×10^{-5}	2.82×10^{-5}
**Ethylene oxide**	8.18×10^{-3}	ND	ND	3.47×10^{-2}	ND	7.27×10^{-3}	1.27×10^{-2}	1.45×10^{-2}
**Formaldehyde**	1.76×10^{0}	1.59×10^{0}	6.10×10^{-1}	1.21×10^{0}	8.15×10^{-2}	8.40×10^{-1}	2.69×10^{-1}	6.16×10^{-1}
**Mercury**	3.30×10^{-4}	2.33×10^{-4}	2.42×10^{-4}	3.10×10^{-4}	3.82×10^{-5}	3.98×10^{-4}	1.27×10^{-4}	1.29×10^{-4}
**Hydroquinone**	4.54×10^{-1}	1.41×10^{-2}	3.91×10^{-1}	1.09×10^{0}	ND	7.94×10^{-2}	2.18×10^{-2}	4.70×10^{-2}
**Isoprene**	2.27×10^{-1}	9.32×10^{-3}	1.07×10^{-1}	3.25×10^{-1}	ND	3.09×10^{-2}	1.45×10^{-2}	9.32×10^{-3}
**m-Cresol**	4.32×10^{-3}	1.36×10^{-3}	3.18×10^{-3}	2.88×10^{-3}	ND	3.64×10^{-3}	1.82×10^{-3}	1.36×10^{-3}
**Methyl ethyl ketone**	1.10×10^{0}	2.50×10^{-1}	6.98×10^{-1}	1.15×10^{0}	ND	8.33×10^{-1}	1.05×10^{-1}	2.50×10^{-1}
**Nickel**	3.61×10^{-3}	3.61×10^{-3}	2.41×10^{-3}	2.41×10^{-3}	3.77×10^{-3}	3.62×10^{-3}	6.89×10^{-4}	3.61×10^{-3}
**Nicotine**	1.39×10^{2}	1.50×10^{2}	1.35×10^{2}	1.72×10^{2}	3.90×10^{1}	9.45×10^{1}	2.36×10^{1}	1.02×10^{2}
**N-Nitroso nornicotine (NNN)**	4.72×10^{-4}	3.92×10^{-3}	3.67×10^{-4}	1.55×10^{-3}	1.82×10^{-5}	2.68×10^{-3}	7.42×10^{-5}	2.18×10^{-3}
**o-Cresol**	7.42×10^{-3}	1.82×10^{-3}	8.59×10^{-3}	5.95×10^{-3}	ND	5.45×10^{-3}	ND	1.82×10^{-3}
**o-Toluidine**	2.12×10^{-4}	2.37×10^{-5}	1.35×10^{-4}	1.37×10^{-4}	ND	1.14×10^{-4}	5.75×10^{-5}	5.65×10^{-5}
**Lead**	1.97×10^{-4}	1.11×10^{-4}	7.42×10^{-5}	2.45×10^{-4}	4.25×10^{-5}	3.69×10^{-4}	7.47×10^{-5}	1.11×10^{-4}
**p-Cresol**	7.73×10^{-3}	2.27×10^{-3}	5.45×10^{-3}	5.15×10^{-3}	ND	3.64×10^{-3}	3.64×10^{-3}	2.27×10^{-3}
**Phenol**	2.00×10^{-1}	5.91×10^{-3}	1.78×10^{-1}	1.70×10^{-1}	9.09×10^{-3}	2.85×10^{-2}	9.09×10^{-3}	5.91×10^{-3}
**Propionaldehyde**	1.56×10^{0}	4.05×10^{-1}	1.16×10^{0}	2.06×10^{0}	ND	9.18×10^{-1}	7.27×10^{-2}	7.59×10^{-1}
**Propylene Oxide**	1.25×10^{-2}	3.55×10^{-3}	9.20×10^{-3}	2.06×10^{-2}	ND	3.55×10^{-3}	5.67×10^{-3}	3.55×10^{-3}
**Pyridine**	1.05×10^{0}	ND	6.34×10^{-1}	9.31×10^{-1}	ND	5.62×10^{-1}	9.09×10^{-3}	ND
**Resorcinol**	3.64×10^{-3}	3.64×10^{-3}	2.42×10^{-3}	8.33×10^{-3}	ND	3.64×10^{-3}	5.45×10^{-3}	3.64×10^{-3}
**Selenium**	1.89×10^{-4}	ND	1.26×10^{-4}	2.38×10^{-4}	ND	1.45×10^{-4}	7.03×10^{-5}	ND
**Styrene**	2.39×10^{-2}	ND	3.05×10^{-2}	1.60×10^{-1}	ND	9.09×10^{-3}	3.64×10^{-3}	ND
**Toluene**	2.25×10^{-1}	4.64×10^{-2}	1.80×10^{-1}	2.75×10^{-1}	ND	5.33×10^{-2}	2.18×10^{-2}	1.39×10^{-2}
**Hydrogen cyanide**	3.98×10^{-1}	1.19×10^{-1}	2.65×10^{-1}	6.62×10^{-1}	ND	2.13×10^{-1}	9.45×10^{-2}	2.12×10^{-1}
**Vinyl chloride**	1.49×10^{-4}	1.49×10^{-4}	9.95×10^{-5}	3.32×10^{-4}	ND	1.49×10^{-4}	2.38×10^{-4}	1.49×10^{-4}
